# HDFT-MViT: a progressive core-enhanced mix framework for Alzheimer’s disease classification using MRI images

**DOI:** 10.3389/fneur.2026.1860368

**Published:** 2026-06-18

**Authors:** Dongyan Zhang, Jincan Zhang, Bo Liu, Min Liu, Wenna Chen, Ganqin Du

**Affiliations:** 1 College of Information Engineering, Henan University of Science and Technology, Luoyang, China; 2 The First Affiliated Hospital, and College of Clinical Medicine of Henan University of Science and Technology, Luoyang, China

**Keywords:** Alzheimer’s disease, dynamic filter, lightweight transformer, magnetic resonance imaging, progressive core-enhanced mixing

## Abstract

**Background:**

Early and accurate diagnosis of Alzheimer’s disease (AD) is critical. In MRI-based computer-aided diagnosis, convolutional neural networks (CNNs) excel at extracting local features but struggle to model long-range dependencies, while Vision Transformers (ViTs) offer strong global modeling capabilities but suffer from high computational complexity, limiting their deployment in resource-constrained settings.

**New methods:**

This paper proposes HDFT-MViT, a lightweight hybrid architecture based on MobileViT that integrates a hierarchical dynamic filter with a lightweight Transformer. The model adopts a progressive Core-Enhanced Mix design: Shallow layers employ MobileNetV2 inverted residual blocks for efficient local feature extraction; intermediate and deep layers incorporate a dual-branch module that integrates a dynamic filter for frequency-domain global modulation and a lightweight Transformer for spatial long-range dependency modeling, followed by hierarchical fusion via learnable weights. A channel attention mechanism is further introduced to enhance feature discriminability.

**Results:**

Evaluations on the public ADNI-1 (3-class) and ADNI-2 (4-class) MRI datasets show that HDFT-MViT achieves state-of-the-art classification accuracies of 98.85 ± 0.27% and 98.07 ± 0.54%, respectively, while maintaining a lightweight profile with only 3.46 M parameters, confirming its effectiveness and efficiency.

**Conclusion:**

HDFT-MViT achieves an optimal balance between local detail perception and global semantic understanding within a computationally efficient framework, offering a promising tool for clinical AD diagnosis. Code will be released upon acceptance.

## 1 Introduction

AD is a progressive neurodegenerative disorder with both hereditary and sporadic forms, characterized by the gradual loss of neurons in the brain, leading to progressive cognitive decline and functional impairment in daily life (1). As the sixth leading cause of death globally and the most common etiology of dementia, AD poses a profound public health challenge, imposing substantial burdens on healthcare systems, affected individuals, and their families (2). MRI serves as a vital tool in early clinical assessment, enabling non-invasive visualization of cerebral structure and detection of subtle soft-tissue alterations associated with AD, thereby facilitating timely diagnosis and intervention (3).

Deep learning has revolutionized computer vision tasks, including image classification and segmentation, opening new avenues for medical image analysis. Among these techniques, CNNs have long served as the cornerstone of medical image interpretation, owing to their exceptional ability to capture local patterns and spatial hierarchies (4). Widely adopted architectures such as ResNet (5) and MobileNet (6) excel at extracting fine-grained texture and morphological details from regions of interest, making them particularly effective for lesion characterization (7). However, due to the inherent locality of convolutional operations, CNNs primarily rely on nearby pixel relationships, which limits their ability to model long-range dependencies across an image—a critical aspect for holistic medical image understanding.

To address the limitations of CNNs in modeling long-range dependencies, the core mechanism of the Transformer model has been successfully introduced into the field of computer imaging (8). Transformer can effectively address the challenges of complex segmentation tasks such as dispersed lesion regions and variable morphology by capturing global contextual information in medical imaging (9). ViT and its derivatives have demonstrated excellent performance in various visual tasks and have been widely used (10). However, the architecture still faces the problem of high secondary computational complexity due to the self-attention mechanism.

To balance global modeling with computational efficiency, researchers turn to Fast Fourier Transform (FFT)-based methods. The global filter realizes attention-like low-pass filtering through frequency domain operations and is more computationally efficient. The FFT-based Dynamic Token Mixer, as proposed by Tatsunami and Taki (11) is able to maintain the competitiveness of the model in visual tasks. Based on this, they further constructed a hybrid model CDFFormer combining convolution and FFT-based dynamic filter, which effectively integrates local feature extraction and global context modeling capabilities, achieving a balance between efficiency and capability.

Based on these, this paper proposes a HDFT-MViT for Alzheimer’s disease medical image classification. Building upon MobileViT framework, the model innovatively introduces a progressive Core-Enhanced Mix that dynamically integrates dynamic filter processing and transformer modeling, achieving an organic unification of local detail perception and global semantic understanding.

Based on the above architecture, this paper proposes a HDFT-MViT model for medical image classification of Alzheimer’s disease. The model innovatively introduces a progressive Core-Enhanced Mix design based on the lightweight MobileViT framework. Through the hierarchical design structure, dual-branch blocks are adaptively fused in the middle and deep layers of the network, thus realizing the integral unity of local detail perception and global semantic understanding.

- Designing a hierarchical five-layer architecture: the shallow layers captures local features with inverted residual blocks, and the middle and deep layers achieve a smooth transition from local to global by dynamic filters with lightweight Transformer for frequency-space domain synergy.
- Building an efficient localized feature extraction module based on MobileNetV2: preserving the inverse residual structure intact in the shallow network, capturing subtle pathological features with low computational overhead, and laying the groundwork for subsequent feature extraction.
- A dual-branch block for hierarchical Dynamic Filter and Transformer fusion: proposing a two-way structure for frequency-domain dynamic filtering in parallel with a lightweight Transformer. Used in the middle and deep layers, hierarchical fusion achieves progressive feature extraction enhancement from local to global.
- The module employs attention mechanisms: Embedded with Efficient Channel Attention (ECA) and Mixed Pooled Channel Attention (MPCA), channel screening and feature enhancement are performed in the pre-processing and post-processing phases, respectively, to improve the model’s perceptual ability and feature discriminative properties of key pathological regions.

## 2 Related work

Over the years, CNNs have shown great potential in medical image classification tasks. For example, Li et al. (12) achieved great success in AD diagnosis using CNNs, which could automatically extract features through end-to-end learning to automatically extract features. He et al. (13) proposed residual connectivity, a structure that transformed a deep network into a shallow network. The design of ResNet has made great strides and has achieved better results in clinically assisted diagnosis of serious diseases such as lung tumors, breast cancer, and skin disease effects (14). DenseNet (15) innovatively proposed feed-forward connections between any two layers with the same feature map size, which facilitated the expansion of the network to hundreds of layers; the evolution of its structure, and the direction of its improvement has been widely used in medical imaging (16). Deep learning has also been widely used in Alzheimer’s image classification. Shamrat et al. (17) proposed a fine-tuned CNN classifier, AlzheimerNet, which was a model trained by CLAHE enhancement and data enhancement, using ADNI data for training better classification results were achieved. Sindhu and Kumaratharan (18) proposed the RP-Net_TaylorDOX-based DNFN method, which integrates a Taylor series-optimized RP-Net for image segmentation and a deep neuro-fuzzy network for grading, achieving automated detection and severity classification of Alzheimer’s disease. Alorf and Khan (19) proposed BC-GCN which considered the brain functional connectivity matrix as a graph and extracted higher-order connectivity features through graph path convolution and edge/node pooling modules to achieve the identification of key brain regions for AD. The CNN methods required combining multiple layers to capture relationships between features through the stacking of convolutional layers, which increased sensitivity to overfitting (20). However, using CNNs with too many layers might result in performance degradation due to redundant information (21). Additionally, the inherent locality of convolutional operations made it difficult to effectively model long-range dependencies—a capability critical for understanding the global contextual information in complex medical images.

The introduction of the Transformer (22) has achieved groundbreaking progress and garnered widespread attention in the field of NLP (23). Its remarkable capability in modeling long-range dependencies has also prompted researchers to successfully adapt and apply its core architecture to computer vision tasks. Lei et al. (24) used Transformer as a base network to extract multi-template regional features for AD recognition, thus capturing rich brain image information. Both ViT and its variants have shown excellent performance and are widely used in mainstream vision tasks (25). Sen et al. (26) proposed a ViT and a Metaheuristic Algorithms for Early AD Detection were comparatively analyzed and better results were obtained. Kumar et al. (27) by iteratively transforming the encoder to form a hierarchical structure with gradually decreasing dimensionality between layers, thereby applying attention mechanisms at different scales to capture local and remote relationships between image blocks. Wang et al. (28) adopted a two-stage framework for compressed WSI classification in order to effectively solve the feature extraction and spatial information loss problems. Swin Transformer (29) used two different slider operations when employing a stage-based design. Nevertheless, the application of Transformer in medical imaging still faces the dependence on large-scale data and arithmetic power due to high computational complexity.

To reduce the computational complexity, some studies have tried to replace self-attention with other global operations. Among them, FFT-based global filters such as GFNet (30) used Fourier Transform with learnable global filters in the frequency domain to mix the information, solving the problem of self-attention and MLP computational complexity increasing with the quadratic growth of image size. Wang et al. (31) addressed the lack of content adaptivity of traditional vision MLPs in token fusion by dynamically generating mixing matrices instead of static MLPs. Rao et al. (32) proposed an adaptive weight mixing that generated attentional weights without inter-label interactions, based on which the generation is able to capture without self-attention both long-term and short-term spatial dependencies without self-attention. Yu et al. (33) proposed a generalized architecture for MetaFormer, replacing the attention mechanism with pooling, proving that the architecture itself was more important than the specific token mixer, and performing well in several vision tasks.

In medical image analysis, capturing both local details and global context is essential. CNNs excel at extracting local features but struggle to model long-range dependencies, while ViTs offer powerful global modeling capabilities yet suffer from high computational complexity and a lack of local inductive bias. Therefore, current research trends favor a synergistic integration of both approaches: leveraging convolutions in shallow layers to capture fine-grained local patterns, and incorporating attention mechanisms in deeper layers to establish long-range semantic relationships.

The proposed HDFT-MViT model embodies this integrative philosophy. Through a progressive hierarchical design, the shallow layers employ MobileNet-style inverted residual blocks for efficient local feature extraction. In the middle to deep layers, a dual-branch block—combining a dynamic filter and a lightweight Transformer—enables collaborative global modulation in the frequency domain and long-range dependency modeling in the spatial domain. This architecture maintains a lightweight footprint while achieving an optimal balance between local detail preservation and global contextual understanding.

## 3 Methods

### 3.1 Proposed approach

**Figure 1:**
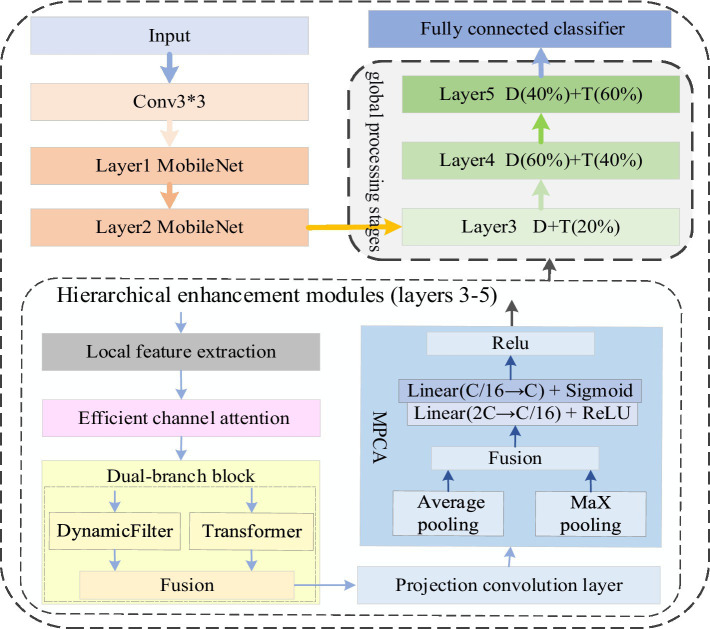
The framework of the HDFT-ViT.

The overall architecture of HDFT-MViT is shown in Figure 1, which adopts a five-layer sequential stacking hierarchical design, corresponding to layer1 to layer5. Each layer takes the output of the previous layer as the input, and gradually completes the feature transformation from spatial details to high-level semantics.

HDFT-MViT adopts a hierarchical progressive architecture. Layers 1 and 2 consist of multiple MobileNet-style inverted residual blocks, which effectively preserve spatial details. Layers 3–5 serve as global processing stages, each following a unified workflow: First, local convolutions extract features, followed by ECA to filter critical channels. Subsequently, a dual-branch block processing module is employed—dynamic filters perform adaptive modulation in the frequency domain, while lightweight Transformers model long-range dependencies in the spatial domain, with progressive fusion via learnable weights. A projection convolution then restores the channel dimension, and MPCA enhances feature representation. Finally, the processed features are concatenated with the input residual and fused via convolution. This design facilitates a hierarchical transition from frequency-domain to spatial-domain global processing, enabling progressive modeling from local details to high-level semantics.

### 3.2 Channel attention mechanism group

In HDFT-MViT, we introduce attention mechanisms: ECA and MPCA, which enhance feature representations through 1D convolutional cross-channel interaction and dual-pooling global statistical fusion, respectively. These two modules are sequentially applied to feature maps to improve the model’s perception of pathological key features.

ECA layer performs channel weight calibration of input features. The module first obtains the channel statistics by compressing the spatial dimensions through adaptive mean pooling. Then, it uses one-dimensional convolution for cross-channel interaction, generates channel weights through sigmoid activation and multiplies them with the original features channel by channel to realize feature enhancement.

As shown in Figure 1, the MPCA module further optimizes the features by using both global average pooling and maximum pooling followed by splicing to generate channel weights through two fully connected layers; finally, it multiplies with the original features and activates them through ReLU to achieve feature enhancement.

### 3.3 Double-branch block

#### 3.3.1 Architectural overview

**Figure 2:**
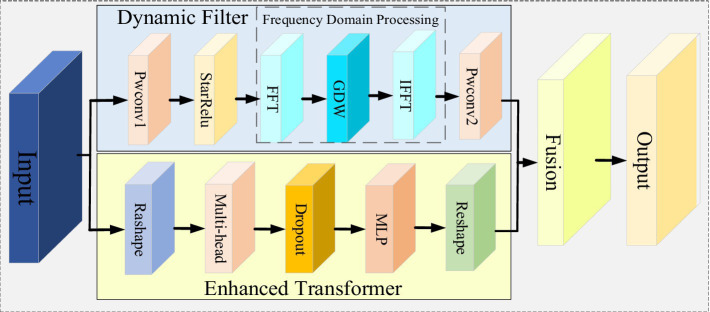
Details of the dual-branch block.

The dual-branch block serves as the core innovative module of this study, as shown in Figure 2: achieving efficient feature extraction through the synergistic design of frequency domain processing and lightweight Transformer enhancement. This module adopts an independent-fusion dual-path design: the dynamic filter path performs feature modulation in the frequency domain, while the Transformer path models global dependencies in the spatial domain. The two paths process from the original input independently and subsequently undergo hierarchical fusion.

#### 3.3.2 Dynamic filter path: frequency domain feature modulation

##### 3.3.2.1 Input representation

Given an input feature tensor X∈ℝ^B × H × W × C^, where *B* denotes the batch size, *H* and *W* represent the height and width of the feature map, respectively, and *C* indicates the number of input channels.

##### 3.3.2.2 Feature projection

Suppose the input features are initially projected to an expanded channel dimension through a linear transformation, as shown in Equation 1:

$$X^{\prime }=\sigma_{1}(W_{1}X+b_{1})$$ (1)

Where, $X^{\prime }\in {\mathbb{R}}^{B\times H\times W\times C^{\prime }}$ , W*_1_* and *b_1_* are learnable parameters of the linear projection layer; *C′ = r.C* represents the expanded channel dimension, with *r* denoting the expansion ratio; *σ1* denotes the StarReLU activation function, defined as StarReLU(x) = s⋅(ReLU(x))^2^ + b, where *s* and *b* are learnable scaling and bias parameters, respectively. This activation function enhances nonlinear expressive capabilities while maintaining computational efficiency.

##### 3.3.2.3 Routing weight generation

Routing weights are dynamically generated based on global feature statistics, enabling adaptive filter selection as shown in Equation 2:

$$a=\text{Reshape}(\text{softmax}(\mathrm{MLP}(\frac{1}{\mathrm{HW}}\sum_{i=1}^{H}\sum_{j=1}^{W}X_{i,j})))$$ (2)

Where $\alpha \in {\mathbb{R}}^{B\times N_{f}\times C^{'}}$ , and MLP denotes a lightweight multi-layer perceptron; N_f_ indicates the number of learnable filters.

Frequency domain filtering:

The projected features are transformed to the frequency domain via 2D real-valued FFT (Equation 3):

$$F=ℱ(X^{\prime })$$ (3)

Where $F\in {\mathbb{C}}^{B\times H\times N_{f}\times C^{'}}$ , and ℂ denotes the complex domain, as the Fourier transform produces complex-valued frequency representations. W_f_ = [W/2] + 1 denotes the reduced width dimension due to the conjugate symmetry property of real-valued FFT.

Frequency-domain modulation based on routing coefficients and learnable complex filters. Formula 4 and 5 is as follows:

$$W_{d}=\sum_{k=1}^{N_{f}}\alpha :,k,⊙W_{f,k}$$ (4)

$$F^{\prime }=F⊙W_{d}$$ (5)

Where, W_f,k_ represents the k-th learnable complex filter; $\alpha \in {\mathbb{R}}^{B\times N_{f}\times C^{\prime }}$ , denotes routing coefficient tensor.

The modulated features are then transformed back to the spatial domain via inverse FFT (Equation 6):

$$Y_{\mathrm{DF}}^{\prime }={ℱ}^{-1}(F^{\prime })$$ (6)

Where, $Y_{\mathrm{DF}}^{\prime }\in {\mathbb{R}}^{B\times H\times W\times C^{\prime }}$ . The features are projected back to the original channel dimension (Equation 7):

$$Y_{\mathrm{DF}}=\sigma_{2}(W_{2}Y_{\mathrm{DF}}^{\prime }+b_{2})$$ (7)

Where, $Y_{\mathrm{DF}}\in {\mathbb{R}}^{B\times H\times W\times C}$ , W_2_ and b_2_ are learnable parameters; *σ_2_* denotes an optional activation function.

#### 3.3.3 Transformer path: spatial global dependency modeling

Feature flattening:

The original input features are flattened for Transformer processing (Equation 8):

$$X_{\text{flat}}=\text{Reshape}(X)$$ (8)

Where, $X_{\text{flat}}\in {\mathbb{R}}^{B\times (H\cdot W)\times C}$ . Lightweight Transformer Encoding: A lightweight Transformer encoder processes the flattened features (Equation 9):

$$Y_{\text{Trans}}^{\prime }=\text{Transformer}(X_{\text{flat}})$$ (9)

Where, $Y_{\text{Trans}}^{\prime }\in {\mathbb{R}}^{B\times (H\cdot W)\times C}$ , the Transformer encoder employs multi-head self-attention and feed-forward networks with residual connections, specifically optimized for efficiency in hierarchical architectures.

Spatial restoration: The processed features are restored to the original spatial dimensions (Equation 10):

$$Y_{\text{Trans}}=\text{Reshape}(Y_{\text{Trans}}^{\prime })$$ (10)

Where, $Y_{\text{Trans}}\in {\mathbb{R}}^{B\times H\times W\times C}$ .

### 3.4 Hierarchical fusion strategy

Layers 1–2 employ the MobileNet architecture for local feature extraction, without involving the dual-branch fusion strategy. In layers 3–5, hierarchical fusion is adopted, where the outputs of the two pathways are adaptively fused using a learnable weight *λ*∈[0,1] (Equation 11). This weight is activated by the Sigmoid function and differentially initialized according to the network depth as shown in Equation 12.

$$\lambda =\text{sigmoid}(\omega_{\text{fusion}})$$ (11)

$$\omega_{\text{fusion}}\{\begin{array}{c} 0.2(\text{layer}3)\\ 0.4(\text{layer}4)\\ 0.6(\text{layer}5) \end{array}$$ (12)

The fusion strategy dynamically adjusts based on network hierarchy:

Layer 3-dynamic filter dominant (Equation 13):

$$Y=Y_{\mathrm{DF}}+\lambda Y_{\text{Trans}}$$ (13)

Layer 4-balanced fusion (Equation 14):

$$Y=(1-\lambda )Y_{\mathrm{DF}}+\lambda Y_{\text{Trans}}$$ (14)

The dual-branch block represents a novel architectural innovation that synergistically combines frequency domain processing with hierarchical Transformer enhancement. By adaptively fusing local feature modulation in the frequency domain with global dependency modeling in the spatial domain, this design achieves superior feature extraction capabilities while maintaining computational efficiency.

## 4 Experiments and analysis

### 4.1 Datasets

To evaluate the effectiveness of the proposed method, this study utilizes brain MRI data from the public Alzheimer’s Disease Neuroimaging Initiative (ADNI) database, which is aimed at developing biomarkers to facilitate the early diagnosis and progression monitoring of AD. Based on the ADNI data, three datasets with different levels of classification complexity were constructed to comprehensively evaluate the classification performance and generalization ability of the model.

#### 4.1.1 Three-class task datasets (ADNI-1)

**Figure 3:**
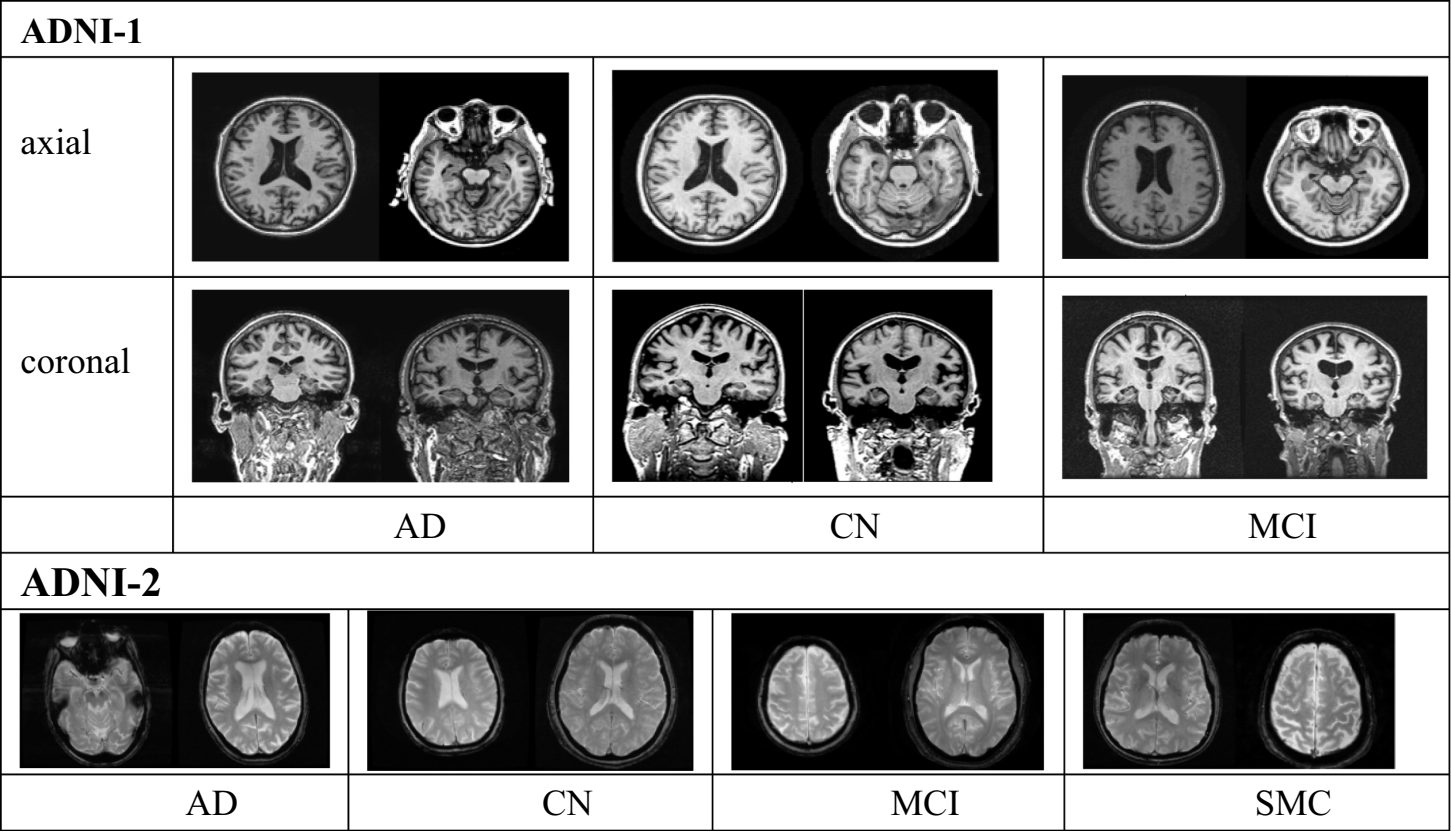
The MRI samples of the ADNI datasets.

The first dataset was constructed from the complete 1-year 1.5 T MRI data in ADNI-1. Subjects with complete baseline scans and well-defined diagnostic labels were selected, resulting in 371 subjects across three categories: AD, Cognitively Normal (CN), and Mild Cognitive Impairment (MCI). The original 3D volumes were stored in NIfTI format. For axial-plane evaluation, the volumes were sliced along the axial direction, preprocessed with window width/level adjustment and normalization, and resized to 256 × 256 pixels in PNG format. Additionally, a coronal-plane version of the same volumes, referred to as ADNI-1-coronal, was generated by reslicing along the coronal direction, producing 4,566 slices from 333 subjects and following the same preprocessing pipeline. For both datasets, a strict subject-level split was applied, with 80% of subjects used for training and 20% for testing. The detailed statistics of the datasets are summarized in Table 1. Furthermore, Figure 3 shows representative MRI slice samples from each diagnostic category in both datasets, providing a visual illustration of the inter-class differences.

**Table 1:** Demographic details of different diagnostic groups in ADNI datasets.

ADNI-1					ADNI-1-coronal		ADNI-2			
Class	Subject	Train images	Test images	Images	Subject	Images	Subject	Train images	Test images	Images
AD	89	1747	437	2,184	84	1,429	79	1,340	336	1,676
CN	116	2,426	607	3,033	116	1,600	88	1,179	334	1,513
MCI	166	3,612	903	4,515	133	1,537	99	1,601	401	2002
SMC	–	–	–	–			63	880	253	1,133
Total	371	7,785	1947	9,732	333	4,566	336	5,000	1,324	6,324

#### 4.1.2 Four-class task dataset (ADNI-2)

To construct a more challenging task that better aligns with fine-grained clinical diagnostic scenarios, we rigorously screened and built a four-class dataset from the ADNI database. This dataset introduces the early-stage category of “Subjective Memory Complaint (SMC),” which, together with AD, CN, and MCI, forms the four classes. The original DICOM files were linearly normalized to a grayscale range of [0, 255] and preprocessed into PNG images of 256 × 256 pixels, with blank slices lacking brain parenchymal tissue being excluded. The data split also followed the subject-level principle, with an 80%/20% ratio allocated to the training set and test set, respectively. This dataset is referred to as ADNI-2 throughout the paper.

### 4.2 Implementation details

All experiments were conducted using Python 3.8, with acceleration provided by a GPU. The implementation utilized widely adopted libraries including TensorFlow, Keras, Scikit-Learn, and NumPy. The hardware configuration used to run the Python-based deep learning framework for Alzheimer’s disease recognition consisted of a system with 32 GB of RAM and an NVIDIA RTX 4060 Ti graphics card equipped with 16 GB of video memory. The design, training, and testing of the models were all performed on a Windows-based operating system.

As shown in Table 2, During training, all input images were uniformly resized to a spatial resolution of 224 × 224 pixels. To ensure the reproducibility of the experimental results, the random seed was fixed to 41 for all stochastic operations. The model was optimized using the AdamW optimizer with an initial learning rate of 1 × 10^−4^ and a weight decay coefficient of 1 × 10^−4^. A cosine annealing learning rate scheduler was adopted to dynamically adjust the learning rate during training. The batch size was set to 8, and the maximum number of training epochs was set to 150. The cross-entropy loss function was employed as the training objective to minimize the classification error between the predicted outputs and the ground-truth labels.

**Table 2:** Parameters used for model training.

Parameters	Settings
Input image size	224 × 224
Random seed	41
Optimizer	AdamW
Learning rate	1e-4
Weight decay	1e-4
Learning rate scheduler	Cosine annealing LR
Batch size	8
Epochs	150
Loss function	Cross-entropy loss
Early stopping patience	15

Furthermore, to mitigate model overfitting and enhance generalization capability, an early stopping mechanism based on validation accuracy was introduced. Specifically, the patience of the early stopping mechanism was set to 15, meaning that training would be terminated early if the validation accuracy did not improve for 15 consecutive epochs, after which the model weights corresponding to the best validation performance would be restored.

### 4.3 Evaluation metrics

In medical image classification tasks, relying on a single evaluation metric is often insufficient to comprehensively assess model performance. To ensure the accuracy and reliability of the evaluation outcomes, this study adopts a set of complementary metrics for a holistic performance analysis. Specifically, accuracy (Acc), precision (Pre), recall (Rec), and the F1-score (F1) are employed to systematically evaluate the classification efficacy of the proposed model. The fundamental outcomes used to assess the classification system consist of four categories: true positives (TP), true negatives (TN), false positives (FP), and false negatives (FN). The computational formulas for these metrics are defined as follows (from formula 15 to 18):

$$\text{Accuracy}=\frac{\mathrm{TP}+\mathrm{TN}}{\mathrm{TP}+\mathrm{TN}+\mathrm{FP}+\mathrm{FN}}$$ (15)

$$\mathrm{Pr}\text{eciosion}=\frac{\mathrm{TP}}{\mathrm{TP}+\mathrm{FP}}$$ (16)

$$\text{Recall}=\frac{\mathrm{TP}}{\mathrm{TP}+\mathrm{FN}}$$ (17)

$$F1‐\text{score}=\frac{2\mathrm{TP}}{2\mathrm{TP}+\mathrm{FP}+\mathrm{FN}}$$ (18)

### 4.4 Results and analysis

#### 4.4.1 Results

All reported metrics are presented as mean ± standard deviation over five independent runs, ensuring robustness and reproducibility. The HDFT-MViT model proposed in this study demonstrates excellent classification performance on both the ADNI-1 and ADNI-2 datasets. As shown in Table 3, on the ADNI-1 dataset, the proposed model achieves an overall classification accuracy of approximately 98%, with precision, recall, and F1-score values consistently exceeding 95% across all categories. In particular, the AD category achieves nearly perfect classification performance, with a precision of 99.88 ± 0.16%, recall of 99.63 ± 0.30%, and F1-score of 99.82 ± 0.18%, indicating the strong capability of the proposed framework in identifying typical Alzheimer’s disease patterns. The CN and MCI categories also demonstrate highly balanced performance, suggesting that the model maintains stable discrimination ability even for intermediate disease stages.

**Table 3:** The prediction results of different methods on the ADNI datasets.

ADNI-1					ADNI-2			
Class	Pre(%)	Rec(%)	F1(%)	Acc(%)	Pre(%)	Rec(%)	F1(%)	Acc(%)
AD	99.88 ± 0.16	99.63 ± 0.30	99.82 ± 0.18		99.94 ± 0.12	99.88 ± 0.16	99.91 ± 0.09	
CN	98.06 ± 0.42	98.35 ± 0.60	98.21 ± 0.38	98.85 ± 0.27	98.17 ± 0.80	96.35 ± 1.35	97.25 ± 0.79	98.07 ± 0.54
MCI	98.83 ± 0.48	98.80 ± 0.34	98.82 ± 0.31		96.58 ± 1.32	99.70 ± 0.37	98.11 ± 0.79	
SMC	–	–	–	–	97.91 ± 0.99	95.34 ± 1.69	96.59 ± 0.70	

On the more challenging ADNI-2 dataset, which includes an additional SMC category and more diagnostically ambiguous cases, the proposed model achieves an overall accuracy of approximately 98%, with precision, recall, and F1-score values for all categories remaining above 95%. Notably, most classification confusion occurs among CN, MCI, and SMC categories, which is clinically understandable because MCI and SMC represent transitional or early-stage cognitive impairment conditions whose neuroanatomical changes often overlap with those of cognitively normal subjects and early AD patients. Nevertheless, the model maintains relatively balanced precision and recall across all categories, indicating that the high overall accuracy is not solely dominated by majority classes.

**Figure 4:**
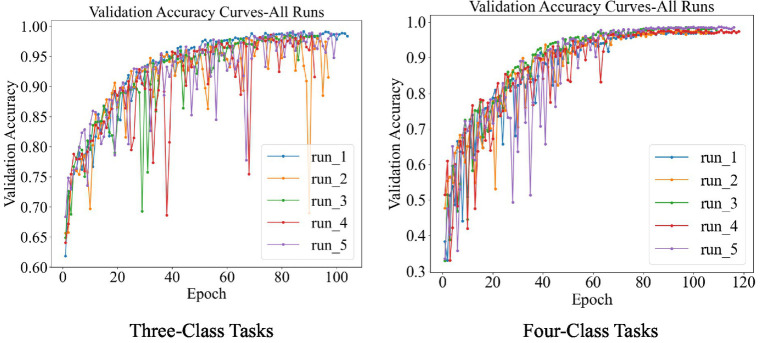
Epoch-wise validation accuracy for five independent runs.

Figure 4 illustrates the validation accuracy curves over epochs for all five independent runs of the proposed model. The left panel corresponds to the three-class task, while the right panel corresponds to the more challenging four-class task. Each colored line represents one of the five runs, showing consistent convergence trends across runs. As observed, the validation accuracy steadily increases with training epochs and stabilizes near the final epochs, indicating that the model training is robust and reproducible across multiple runs. Notably, the curves for the four-class task show slightly more fluctuation in early epochs, reflecting the increased difficulty of distinguishing among diagnostically adjacent categories, yet all runs converge to high validation accuracy by the end of training.

**Figure 5:**
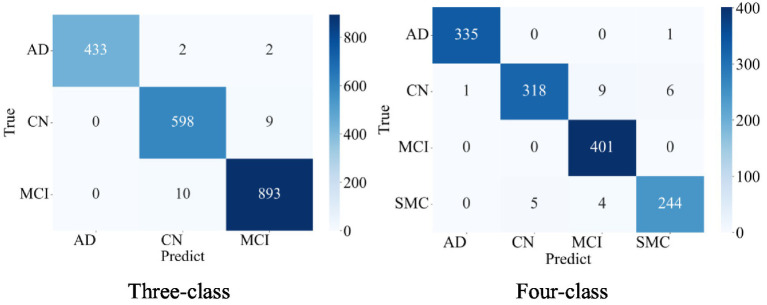
The test confusion matrix.

In addition, the confusion matrices shown in Figure 5 demonstrate that the diagonal elements are highly concentrated, indicating that the proposed model achieves strong discriminative capability across different diagnostic categories in both the three-class and four-class classification tasks. Most samples are correctly classified into their corresponding categories, reflecting the effectiveness of the proposed hierarchical feature fusion framework in capturing disease-related structural characteristics.

For the three-class classification task, only a small number of misclassifications occur between CN and MCI categories. This phenomenon is clinically understandable because MCI represents a transitional stage between normal cognitive aging and Alzheimer’s disease, and its neuroanatomical changes are often subtle and partially overlap with those of cognitively normal subjects. Similarly, in the four-class classification task, most prediction errors are concentrated among CN, MCI, and SMC categories, while AD samples are classified with relatively high accuracy. In particular, several SMC samples are misclassified as CN or MCI, which may be attributed to the highly ambiguous and early-stage characteristics of subjective memory complaints. Since SMC and early MCI patients often exhibit mild or inconspicuous structural brain alterations, the corresponding MRI features may not yet show sufficiently distinct pathological patterns.

**Figure 6:**
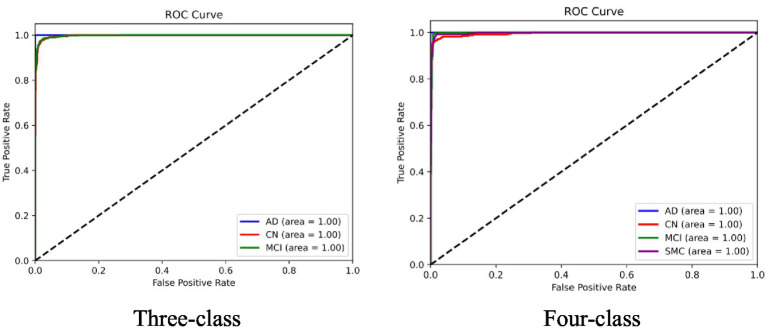
The ROC curves of the proposed model.

As shown in Figure 6, the ROC curves show the results of the proposed model, indicating that the model exhibits excellent performance, which is consistently located in the upper left region of the graph.

These results further indicate that the proposed model maintains robust classification performance even in diagnostically challenging and clinically overlapping categories. At the same time, they also highlight the inherent difficulty of distinguishing subtle transitional stages in Alzheimer’s disease progression, which remains a common challenge in medical image-based AD classification studies.

**Figure 7:**
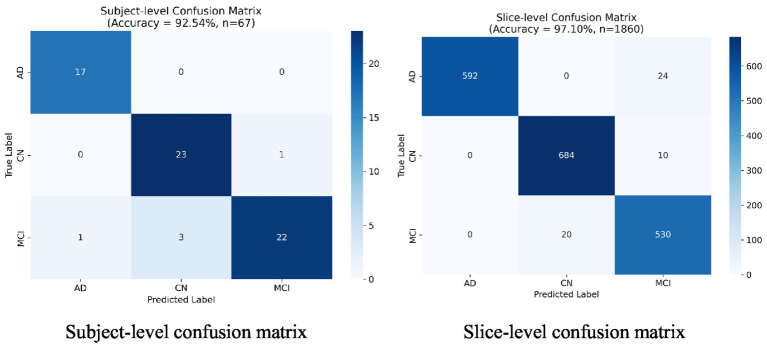
Subject-level and slice-level confusion matrices for the three-class.

Although subject-level train/test splitting is applied, evaluation at the slice level can artificially inflate performance metrics due to intra-subject correlations. Figure 7 presents both subject-level (left) and slice-level (right) confusion matrices, while Table 4 summarizes precision, recall, F1-score, and accuracy for each class.

**Table 4:** Subject-level and slice-level classification metrics.

ADNI-1-cornal	Subject-level				Slice-level			
Class	Pre(%)	Rec(%)	F1(%)	Acc(%)	Pre(%)	Rec(%)	F1(%)	Acc(%)
AD	94.44	100.00	97.14		100.00	96.10	98.01	
CN	88.46	95.83	92.00	92.54	97.16	98.56	97.85	97.10
MCI	95.65	84.62	89.80		93.97	96.36	95.15	

At the subject level, the proposed model achieves an overall accuracy of 92.54%, with class-wise precision (94.44%), recall (100.00%), and F1-score (97.14%). In contrast, slice-level evaluation yields a higher overall accuracy of 97.10%, indicating that slice-level metrics may overestimate diagnostic performance. Therefore, subject-level aggregation is necessary to accurately reflect the model’s true discriminative capability across individuals.

**Figure 8:**
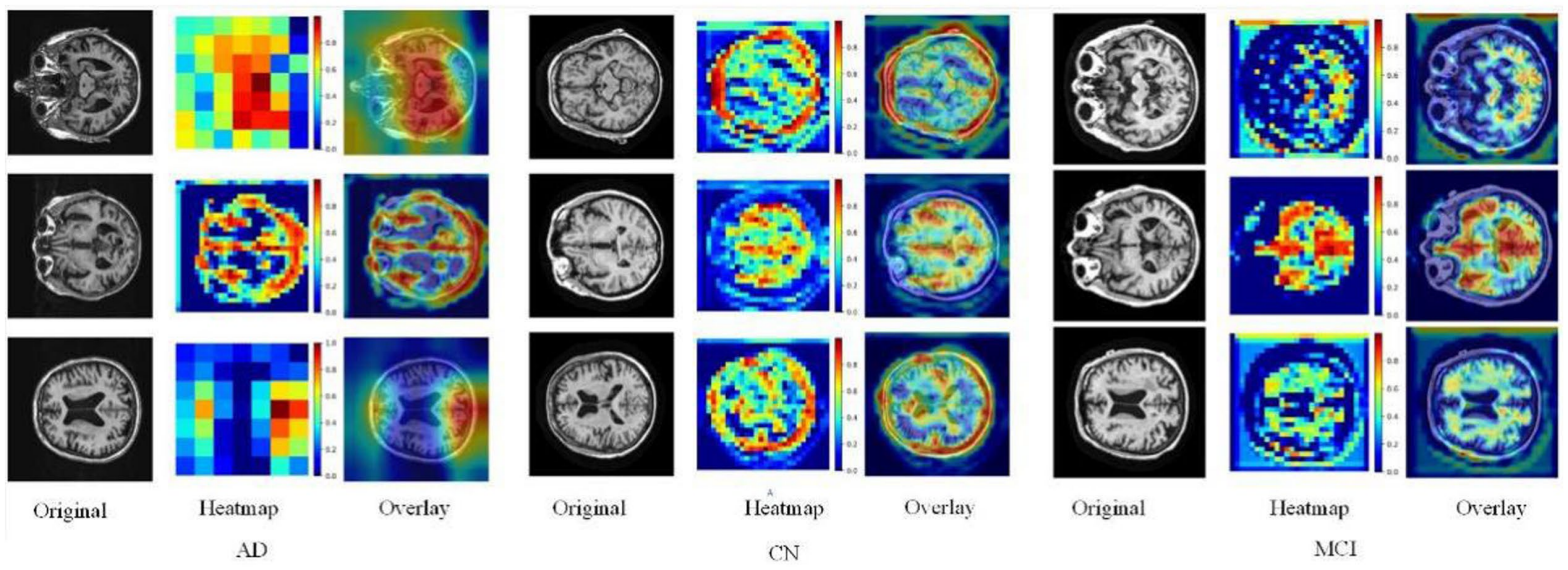
Heatmaps generated by the proposed HDFT-MViT on sample AD MRI images.

To explain the high performance of HDFT-MViT in MRI classification of Alzheimer’s disease, this study used Grad-CAM to generate heat maps for analyze the model’s attention distribution. The Figure 8 shows that for the AD samples, the model attention was concentrated in atrophic regions such as the cerebral cortex and hippocampus, consistent with typical pathological features; for the CN samples, the attention was uniformly distributed; and for the MCI samples, the attention extensively covered the hippocampus and the peripheral cortex, reflecting the possible diffuse changes of MCI. This suggests that HDFT-MViT can adaptively focus on diagnostically critical regions, and its attentional pattern is consistent with the clinical *a priori*, which enhances the interpretability of the model and the credibility of the assisted diagnosis.

The excellent performance of the proposed model primarily stems from its progressive hierarchical fusion architecture. This architecture efficiently extracts local features in layers 1–2 using MobileNet-style inverted residual blocks, laying a detailed foundation for subsequent frequency-domain and Transformer-based feature refinement. In layers 3–5, the model incorporates a core dual-branch global processing block that adaptively fuses an FFT-based dynamic filter with a lightweight Transformer. This design facilitates a smooth transition from frequency-domain-dominated global processing to spatial-domain-dominated global modeling, establishing a progressive feature learning pathway from local detail perception to high-level semantic understanding. Furthermore, Attention mechanism groups collaborate in tandem to achieve multilevel feature optimization of channel dimensions in a hierarchical structure, which together enhance feature discriminative and spatial perceptual capabilities. Overall, this hierarchical fusion strategy enables the model to adaptively balance the preservation of local details with the modeling of global context, thereby strengthening its capability to capture intricate pathological patterns in Alzheimer’s disease MRI images and achieve high classification accuracy.

#### 4.4.2 Ablation study

To validate the effectiveness of each core module in HDFT-MViT, we conducted systematic ablation studies. By progressively removing or combining the Efficient Channel and Coordinate Attention mechanisms (A), the Dynamic Filter (D), and the Lightweight Transformer (T), we quantitatively evaluated their contributions to model performance.

As shown in Table 5, the HDFT-MViT (A + D + T) achieved the best accuracy on both three-class and four-class tasks (98.82 and 98.04%, respectively), demonstrating the necessity of multi-component synergy. Individual modules performed inadequately: using only Attention (A) resulted in insufficient global modeling capability; using only the Dynamic Filter (D) led to weak feature selection; and using only the Transformer (T) underutilized local and frequency-domain information. The combination of Dynamic Filter and Transformer (D + T) performed close to the full model on the three-class task (98.72%), indicating functional complementarity. However, its performance significantly dropped on the more complex four-class task (97.28%), highlighting the critical role of the attention mechanism in feature optimization.

**Table 5:** The ablation experiment results.

Number of classes	A	D	T	Pre(%)	Rec(%)	F1(%)	Acc(%)
Three	√			97.41	96.93	97.15	96.87
		√		97.24	97.67	97.42	97.12
			√	97.73	97.80	97.77	97.59
	√	√		97.62	97.01	97.29	97.02
	√		√	96.90	96.67	96.78	96.46
		√	√	98.85	98.80	98.82	98.72
	√	√	√	98.94	98.83	98.88	98.82
Four	√			96.46	95.48	95.87	95.92
		√		97.27	96.98	97.08	97.13
			√	97.30	97.03	97.14	97.28
	√	√		97.23	96.80	97.00	97.21
	√		√	97.58	97.04	97.28	97.43
		√	√	97.59	97.20	97.34	97.28
	√	√	√	98.06	97.84	97.93	98.04

The results indicate that the performance advantage of HDFT-MViT stems from the organic synergy of Attention, Dynamic Filter, and Transformer: Attention optimizes feature selection, the Dynamic Filter enables efficient frequency-domain fusion, and the Transformer models long-range dependencies. Together, they form a lightweight yet powerful classification framework.

#### 4.4.3 Comparison with classical models

To comprehensively evaluate the performance of the proposed HDFT-MViT model, we conducted comparative experiments against a series of classical and state-of-the-art deep learning models on the same ADNI-1 (three-class) and ADNI-2 (four-class) datasets. The compared models encompass a wide spectrum of architectures, ranging from early foundational designs to recent efficient lightweight networks, including AlexNet, ConvNeXt, DenseNet, EfficientNetV2, ShuffleNet, ResNet50, MobileNetV2, MobileNetV3, and the original MobileViT.

As summarized in Table 6, HDFT-MViT achieves the best overall performance on both classification tasks. In the three-class task, it leads all competitors across all metrics: accuracy (98.82%), precision (98.94%), Recall (98.83%), and F1-score (98.88%). It demonstrates a marginal yet consistent improvement over the next best performers, ResNet50 (98.66% accuracy) and EfficientNetV2 (98.66% accuracy). More notably, compared to other lightweight models designed for efficiency, such as MobileNetV3 (96.82% accuracy) and the original MobileViT (98.46% accuracy), HDFT-MViT shows a more pronounced advantage, surpassing them by 2.00 and 0.36 percentage points in accuracy, respectively. This trend holds for the more challenging four-class task, where HDFT-MViT maintains its lead with 98.04% accuracy, significantly outperforming ResNet50 (97.43%) and MobileNetV3 (94.86%). To ensure the fairness of the comparisons, all comparison models have been properly parameterized and adapted.

**Table 6:** The prediction results of different methods.

Model	Class-3				Class-4				Para(M)
	Pre(%)	Rec(%)	F1(%)	Acc(%)	Pre(%)	Rec(%)	F1(%)	Acc(%)	
Alexnet	96.89	96.91	96.90	96.56	92.99	91.91	92.34	92.60	14.59
ConvNext	81.48	80.57	80.92	80.17	74.76	74.27	74.41	74.92	49.46
Densenet	97.87	97.79	97.83	97.59	97.82	97.09	97.40	97.51	6.96
EfficientNetv2	98.79	98.75	98.77	98.66	97.76	97.21	97.45	97.51	20.18
Shufflenet	89.71	88.76	89.13	88.14	72.62	70.85	71.20	72.43	0.34
Resnet50	98.92	98.64	98.77	98.66	97.68	97.00	97.30	97.43	23.51
MobileNetV2	97.61	97.57	97.59	97.33	96.11	95.45	95.74	96.00	2.23
MobileNetV3	97.26	96.98	97.12	96.82	95.29	94.40	94.78	94.86	1.52
MobileViT	98.77	98.46	98.61	98.46	97.37	96.87	97.09	97.28	4.94
Ours	98.94	98.83	98.88	98.82	98.06	97.84	97.93	98.04	3.46

Particularly noteworthy is the favorable balance HDFT-MViT strikes between efficiency and performance. With only 3.46 M parameters, it remains highly efficient. While its parameter count is higher than the extremely compact MobileNetV3 (1.52 M), it is substantially lower than heavyweight models like ResNet50 (23.51 M) and EfficientNetV2 (20.18 M). This indicates that the performance gains of HDFT-MViT are not merely a result of increased model capacity but are primarily attributed to its innovative architectural design.

The performance advantage of HDFT-MViT stems from its innovative progressive hierarchical fusion architecture. The architecture utilizes efficient convolutions in shallow layers to extract local details, while in middle-to-deep layers, it adaptively coordinates an FFT-based dynamic filter with a lightweight Transformer through learnable fusion weights. The dynamic filter achieves data-dependent global context modulation in the frequency domain with near-linear computational complexity, whereas the Transformer models long-range dependencies in the spatial domain. This progressive collaboration—from local to global, and from frequency domain to spatial domain—enables the model to comprehensively and efficiently capture cross-scale pathological patterns in brain MRI scans, thereby achieving high-precision and robust fine-grained classification while maintaining a lightweight structure.

### 4.5 Comparative analysis with other state-of-the-art techniques

To highlight the advantages of our model, we compared it with other prominent models. Table 7 presents a comparative evaluation of the classification accuracy between the proposed model and recent state-of-the-art approaches.

**Table 7:** Comparative analysis with the state-of-the-art techniques.

Number of classes	References	Model	Pre(%)	Rec(%)	F1(%)	Acc(%)
Three	Jomeiri (34)	RAE-ViT	–	–	–	94.2
	Chen (35)	MMDF	96.98	96.40	96.69	97.65
	Haq (36)	CNN-LSTM	92.1	-	92.25	92.3
	Muksimova (37)	Advanced CNN	–	–	–	98.4
	Ours	HDFT-MViT	98.94	98.83	98.88	98.82
Four	Velu (38)	CNN–Swin transformer	95.0	95.0	95.0	95.34
	Choudhury (39)	Coupled-GAN	95	–	94	94.5
	Pruthviraja (40)	GoogleNet model	–	–	–	98
	Helaly (41)	2D-M^2^IC	95	94	95	93.6
	Ours	HDFT-MViT	98.06	97.84	97.93	98.04

On the three-class task, HDFT-MViT achieved an accuracy of 98.82%, surpassing most existing methods. For instance, it outperformed the RAE-ViT model introduced by Jomeiri et al. (94.2%) and the CNN-LSTM framework employed by Haq et al. (92.3%). Even when compared to the high-performing MMDF model proposed by Chen et al. (97.65%), HDFT-MViT demonstrated a noticeable improvement in accuracy.

For the more challenging four-class task, HDFT-MViT maintained its competitiveness with an accuracy of 98.04%. Its performance exceeded that of the CNN-Swin Transformer hybrid model by Velu et al. (95.34%) and the method by Choudhury et al., which utilizes a coupled Generative Adversarial Network for feature fusion (94.5%). A clear advantage was also observed over the 2D-M^2^IC framework proposed by Helaly et al. (93.6%). The GoogLeNet-based model reported by Pruthviraja et al. showed a comparable accuracy of 98%; however, specific task configurations and data details were not fully disclosed, making a direct and precise comparison difficult.

This broader comparison underscores the effectiveness of our proposed method and its potential advantages in Alzheimer’s disease recognition.

## 5 Conclusion

To address the challenges of balancing global and local feature modeling while maintaining computational efficiency in AD classification, this paper has proposed the HDFT-MViT. The model adopts a progressive hierarchical fusion strategy: inverted residual blocks in shallow layers efficiently capture local details; FFT-based dynamic filters in middle layers perform global frequency-domain modulation; and lightweight Transformers in deep layers model long-range spatial dependencies. Additionally, ECA and CA (with mixed pooling) are integrated to enhance feature discriminability through complementary channel attention mechanisms.

Nevertheless, several limitations still exist in the current study. First, all experiments were conducted solely on the ADNI dataset, which may exhibit relatively homogeneous imaging protocols and subject distributions. Consequently, the cross-dataset generalization capability of the proposed framework remains to be further validated on independent multi-center cohorts and real-world clinical datasets. Second, diagnostically ambiguous categories such as MCI and SMC remain challenging due to overlapping neurodegenerative characteristics and subtle structural differences. In addition, potential class imbalance among different diagnostic groups may still affect model discrimination performance for minority categories.

In the future, the present hierarchical fusion framework could be extended to 3D multimodal data, validated in larger scale clinical data, and applied to tasks such as disease prediction and lesion segmentation. In conclusion, HDFT-MViT not only demonstrates high accuracy and robustness in AD classification tasks but also maintains a lightweight architecture suitable for resource-constrained clinical deployment. This study provides an efficient and interpretable progressive dual-branch block for medical image classification, offering valuable insights for future research in early AD diagnosis and other fine-grained medical imaging analysis tasks.

## Data Availability

Publicly available datasets were analyzed in this study. This data can be found at: https://adni.loni.usc.edu/data-samples/adni-data/.
